# Current Progress in the Endogenous Repair of Intervertebral Disk Degeneration Based on Progenitor Cells

**DOI:** 10.3389/fbioe.2020.629088

**Published:** 2021-01-22

**Authors:** Yanbin Zhang, Yiqiang Hu, Wentian Wang, Zijun Guo, Fan Yang, Xianyi Cai, Liming Xiong

**Affiliations:** Department of Orthopaedics, Tongji Medical College, Union Hospital, Huazhong University of Science and Technology, Wuhan, China

**Keywords:** intervertebral disk – degeneration, endogenous repair strategy, microenvironment, progenitor/stem cell, intercellular communication

## Abstract

Intervertebral disk (IVD) degeneration is one of the most common musculoskeletal disease. Current clinical treatment paradigms for IVD degeneration cannot completely restore the structural and biomechanical functions of the IVD. Bio-therapeutic techniques focused on progenitor/stem cells, especially IVD progenitor cells, provide promising options for the treatment of IVD degeneration. Endogenous repair is an important self-repair mechanism in IVD that can allow the IVD to maintain a long-term homeostasis. The progenitor cells within IVD play a significant role in IVD endogenous repair. Improving the adverse microenvironment in degenerative IVD and promoting progenitor cell migration might be important strategies for implementation of the modulation of endogenous repair of IVD. Here, we not only reviewed the research status of treatment of degenerative IVD based on IVD progenitor cells, but also emphasized the concept of endogenous repair of IVD and discussed the potential new research direction of IVD endogenous repair.

## Introduction

Low back pain (LBP) associated with intervertebral disk (IVD) degeneration often prevents patients from participating in the labor market, which brings a very large economic burden to their families and society (Sakai and Grad, 2015; Deyo, 2017). The current clinical treatment for diseases related to IVD degeneration including conservative treatment and surgical treatment cannot completely restore the structural and biomechanical functions of the IVD tissue (An et al., 2003; Bron et al., 2009). Due to the limitations of these clinical treatments, regenerative medicine becomes a promising method for the treatment of IVD degeneration.

**FIGURE 1:**
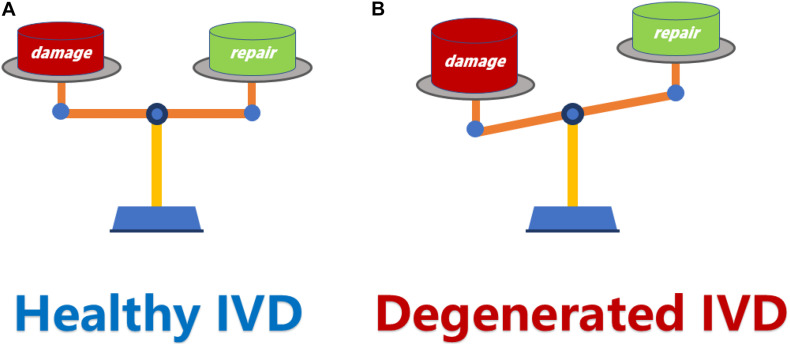
**(A)** The dynamic balance between damage and repair is a necessary condition for maintaining a healthy IVD. **(B)** When the damage exceeds the repair capacity of IVD, the balance between damage and repair is disrupted, and resulting in IVD degeneration. IVD, intervertebral disk.

The essence of IVD degeneration is due to the imbalance between repair and damage (Figure 1). This imbalance is mainly manifested as a relative decrease in anabolism and a relative increase in catabolism in IVD, which leads to the alteration of composition in extracellular matrix (ECM) and severe loss of resident nucleus pulposus (NP) cells and eventually lead to the IVD degeneration (Clouet et al., 2009).

Before IVD progenitor cells were discovered, repairing degenerated IVD by transplanting cells with potential repair effects, such as NP cells and exogenous progenitor/stem cells partially confirmed the feasibility of cell regeneration therapy for degenerative IVD and provided a potential way to prevent degeneration of IVD (Orozco et al., 2011; Cai et al., 2015). However, the use of exogenous cells to treat degenerated IVDs has revealed some biological and ethical limitations.

Intervertebral disk endogenous repair is an effective repair mechanism inside the IVD that allows IVD tissue to remain in a fully functional state for many years after adulthood (Ma et al., 2019). Tissue-specific progenitor cells, known as endogenous progenitor cells, exist in a variety of tissues (Becerra et al., 2011; Yin et al., 2013; Lee C.H. et al., 2015). There are also endogenous progenitor cells in the IVD, which play an important role in the process of endogenous repair. The discovery of IVD progenitor cells has assisted in deepening our understanding of the endogenous repair of IVD and offering potential means to regulate endogenous repair (Risbud et al., 2007; Henriksson et al., 2009; Blanco et al., 2010; Erwin et al., 2011; Sakai et al., 2012; Brisby et al., 2013; Navaro et al., 2015; Sang et al., 2016). Nevertheless, spontaneous repair of IVD progenitor cells does not seem to prevent the IVD degeneration as well as expected, which has been considered as a major cause of IVD degeneration (Silva-Correia et al., 2013; Sakai and Andersson, 2015). On one hand, the main reason for the failure of endogenous repair is that it cannot recruit enough progenitor cells to repair damaged IVD. On the other hand, the adverse microenvironment in degenerative IVD (such as hypoxia, low pH, high mechanical load, inflammation, *etc*.) will seriously affect the viability of progenitor cell within IVD, which prevents them from participating in the repair of degenerative IVD (Risbud et al., 2010; Mizrahi et al., 2013; Jin et al., 2014; Ouyang et al., 2017). Therefore, how to recruit a sufficient number of progenitor cells and maintain the health of them under adverse microenvironments is a significant problem to be solved.

In this review, we summarize current strategies of the regulation of IVD microenvironment and the recruitment of progenitor cell. Besides, we also emphasize some concepts about the IVD endogenous repair and discuss some potential research hotspots of IVD endogenous repair.

## Safety Issues and Concerns of Exogenous Cell-Based Therapies

The treatment of degenerated IVD by supplementing exogenous cells, especially exogenous progenitor/stem cells, has achieved a number of encouraging results and promoted the development of cell therapy for degenerated IVD to an extent (Sakai and Andersson, 2015; Sun et al., 2015). Although many studies have shown that exogenous progenitor/stem cell transplantation can significantly affect the treatment of IVD degeneration (Hiyama et al., 2008; Murrell et al., 2009; Chun et al., 2012; Liang et al., 2013), there are some limitations. First, it is difficult to justify the repeated implantation. Transplantation is an invasive method that may cause iatrogenic injury to the patients and exacerbate the IVD degeneration (Michalek et al., 2010). Secondly, most cells used in transplantation currently come from laboratories, where they require a series of biological procedures such as cell isolation, expansion, freezing and thawing. Each experimental step causes potential risk to biological safety, which will lead to unpredictable harm in patients. Thirdly, there is no method for the replenishment of progenitor cells in the long-term, thus, the maintenance of long-term efficacy is a challenge. Moreover, the high cost of cell transplantation is also a problem that has to be considered.

In terms of the biosecurity, Xu et al. (2009) have transplanted human bone marrow mesenchymal stem cells (BMSCs) into nude mice and found that the cells could promote osteosarcoma growth and metastasis. Similarly, Kamat et al. (2015) have found that human adipose tissue-derived progenitor cells (ADPCs) transplanted into mice promoted the progression and metastasis of tumors by altering their phenotype. In spite of the fact that these evidences are not enough to confirm that the transplantation of exogenous progenitor cell will definitely promote the development of tumors, the safety of exogenous cell transplantation should not be ignored. Additionally, Vadala et al. (2012) have used rabbits to construct an *in vivo* model of degenerated IVD, injecting rabbit BMSC cultured *in vitro* for 3 weeks into the degenerated IVD. The X-ray and MRI results after 3 and 9 weeks do not demonstrate any regeneration of the degenerated IVDs. Instead, a large number of osteophytes have formed on the anterolateral side of the injected IVD following differentiation of the transplanted cells. This suggests that, not only is there risk in the simple procedure of transplanting cells into IVDs where implanted cells can leak out through the cracked fibrous ring due to the high-pressure environment in IVD, but also a risk of unintended differentiation into other cell types. Moreover, Huang Y.Z. et al. (2013) have found that after continuous culture *in vitro*, rat BMSC will spontaneously calcify, while goat and human BMSC cultured will not calcify under the same conditions. Thus, this may indicate that not all species of BMSC are at risk of spontaneous calcification during continuous culture *in vitro*. From a rigorous point of view, we cannot compare the two studies directly. But these biosafety problems show that exogenous cell transplantation has many risks that are currently unpredictable.

In terms of the practical applications, while studying the status of cells following transplantation, it has been found that the majority of transplanted cells remained for a long time in the region where they have been transplanted rather than being actively involved in the repair process (Henriksson et al., 2012). Moreover, studies have shown that the adverse microenvironment in degenerated IVD may lead to death of the transplanted cells (Acosta et al., 2011; Huang Y.C. et al., 2013). Apparently, this suggests that when using cell transplantation to treat degenerative IVD, the method of cell transplantation, the transplantation site and the state of the transplanted cells after transplantation should be considered.

## Endogenous Repair: A New Choice for Cell-Based Therapies

### Resident IVD Progenitor Cells

It has been reported that there are progenitor cells in NP, AF, and CEP tissues and based on the different derived cells, IVD progenitor cells are generally divided into three types: nucleus pulposus-derived progenitor cells (NPPCs), annulus fibrosus-derived progenitor cells (AFPCs) and cartilage endplate-derived progenitor cells (CEPCs) (Risbud et al., 2007; Blanco et al., 2010; Huang et al., 2012). Similar to many endogenous progenitor cells, IVD progenitor cells can also maintain a dynamic balance between repair and damage by regulating their proliferation and differentiation.

Intervertebral disk progenitor cells have many similarities with MSCs in surface markers (Li et al., 2015). The current characterization and related study findings of IVD progenitor cells are shown in the Table 1. Notably, there is currently no standardized international consensus on the surface markers of IVD progenitor cells. Not only that, it has been reported that the IVD cells also have surface markers similar to MSCs, which brings difficulties to the identification of IVD progenitor cells. For instance, Feng et al. (2010) have reported that AF cells have many of the same surface markers as MSC, and Sakai et al. (2012) have shown that human-derived NP cells can also express CD44, CD73, CD90, and CD105 significantly. Therefore, it is necessary to explore the distinct surface markers of IVD progenitor cells. Currently, Sakai et al. (2012) have indicated that the tyrosine kinase endothelial receptor (Tie2) and disialoganglioside 2 (GD2) are sensitive markers of NPPCs, and Chen et al. (2016) have found that the NPPC harvested by differential adhesion method display a higher positive rate of progenitor cell surface markers than NPC. Besides, Erwin et al. (2013) have shown that NPPCs have higher Nanog expression than BMSC.

**TABLE 1:** Characterization and function of intervertebral disk (IVD) progenitor cells.

**Year**	**Cell type**	**Surface positive marker (+)**	**Surface negative marker (−)**	**Key finding**	**References**
**Human**					
2019	NPPC	CD24, CD73, CD90, CD105	CD29, CD45	MSC-CM can alleviate HG induced extracellular matrix degradation via the p38 MAPK pathway and resume the collagen II and aggrecan expression in NPPC	Qi et al., 2019
2018	CEPC	CD73, CD90, CD105	CD14, CD19, CD34, CD45, HLA-DR	BNIP3 contributed to the regulation of cyclic stretch-induced apoptosis of CEPC in an in vitro model	Yuan et al., 2018
2017	NPPC	CD73, CD90, CD105	CD34, CD45, HLA-DR	1. Acidic condition could inhibit NPPC proliferation, extracellular matrix synthesis and the expression of stem cell-related genes and increase cell apoptosis and the expression of ASICs 2. Amiloride can non-specifically inhibit ASCIs	Liu et al., 2017
2016	NPPC	CD73, CD90, CD105	CD34, CD45	NPPC harvested by differential adhesion method display a higher positive rate of progenitor cell surface markers than NPC	Chen et al., 2016
2012	NPPC	Tie2, GD2, Flt1, CD271	CD24	Tie2 and GD2 are sensitive markers of NPPCs	Sakai et al., 2012
2012	CEPC	CD44, CD73, CD90, CD105, CD133, CD166, Stro-1	CD14, CD19, CD34, CD45, HLA-DR	The presence of progenitor cells in degenerated CEP	Huang et al., 2012
2011	CEPC	CD44, CD73, CD90, CD105, CD133, CD166, Stro-1	CD14, CD19, CD34, CD45, HLA-DR	1. CEPC can be induced into osteoblasts, adipocytes, and chondrocytes 2. Compared with BMSC, CEPC are superior in terms of osteogenesis and chondrogenesis	Liu et al., 2011
2010	NPPC	CD90, CD73, CD105, CD106, CD166	CD14, CD19, CD24, CD34, CD45, HLA-DR	The NP contains progenitor cells, which are similar to BMSC	Blanco et al., 2010
2010	AFPC	CD29, CD49e, CD51, CD73, CD90, CD105, CD166, CD184, CD24, Stro-1, nestin, neuron-specific enolase	CD31, CD34, CD45, CD106, CD117, CD133	AFPC have the ability to differentiate into adipocytes, chondrocytes, osteoblasts, neurons and endothelial cells	Feng et al., 2010; Li et al., 2015
2007	AFPC, NPPC	CD49a, CD63, CD73, CD90, CD105, CD166, p75 NTR, CD133/1	CD34	The pathologically degenerate human disk contained populations of skeletal progenitor cells	Risbud et al., 2007
**Rat**					
2017	NPPC	CD73, CD90, CD105	CD34, CD45	The increased senescence of NPPC is present in IVD with advancing age	Zhao et al., 2017
2015	NPPC	CD44, CD90, CD105	CD34, CD45	The synergy between TGF-β3 and IGF-1 ameliorates NPPC viability and differentiation and promotes IVD regeneration	Tao et al., 2015
2009	AFPC	Notch1, Delta4, CD117	Jagged1	The stem cell niche pattern plays a role for IVD morphology and function	Henriksson et al., 2009; Li et al., 2015
**Rabbit**					
2018	AFPC	CD29, CD44, CD166, Oct4, Nucleostemin, SSEA-4	CD4, CD8, CD14	AFPC has the ability to differentiate into adipocytes, osteocytes, and chondrocytes	Guo et al., 2018
2013	NPPC, AFPC	**NPPC**, **AFPC:** PCNA, CD166, C-kit, Jagged1, Notch1	(−)	There are progenitor cells in rabbit IVD, and their number decreases with age	Yasen et al., 2013; Li et al., 2015
2009	NPPC, AFPC	**NPPC:** Delta4, Jagged1, Stro-1 **AFPC:** Notch1, Delta4, Jagged1, CD117, Stro-1, Ki-67	**NPPC:** Notch1, CD117, Ki67 **AFPC:** (−)	The stem cell niche pattern plays a role for IVD morphology and function	Henriksson et al., 2009; Li et al., 2015
**Rhesus macaque**					
2013	NPPC, AFPC	**NPPC:** CD44, CD90, CD146, CD166, HLA-DR **AFPC:** CD44, CD90, CD146, CD166, HLA-DR	**NPPC:** CD90, CD271 **AFPC:** CD29, CD106, CD271	Endogenous progenitor cell population exists in healthy IVD, which is similar in characteristics to the progenitor-like population reported in degenerated IVD	Huang S. et al., 2013
**Dog**					
2013	NPPC	Sox2, Oct3/4, Nanog, CD133, Nestin, neural cell adhesion molecule	Protein 0, Brachyury	NPPCs have higher Nanog expression than BMSC	Erwin et al., 2013
**Pig**					
2009	AFPC	Notch1, Delta4, Jagged1, CD117, Stro-1, Ki-67	(−)	The stem cell niche pattern plays a role for IVD morphology and function	Henriksson et al., 2009

*MSC, mesenchymal stem cells; BMSC, bone marrow-derived mesenchymal stem cells; CEPC, cartilage endplate-derived progenitor cells; NPPC, nucleus pulposus-derived progenitor cells; AFPC, annulus fibrosus-derived progenitor cells; HG, high glucose; MSC-CM, MSC conditioned medium; NP, nucleus pulposus; NPC, nucleus pulposus cells; CEP, cartilage endplate; BNIP3, adenovirus E1B 19 kDa interacting protein 3; IVD, intervertebral disk, ASICs, acid-sensing ion channels.*

Intervertebral disk progenitor cells also have many similarities with MSCs in some biological behaviors. For instance, Huang S. et al. (2013) have found that endogenous progenitor cell population exists in healthy IVD, which is similar in characteristics to the progenitor-like population reported in degenerated IVD, Li X.C. et al. (2017) have demonstrated that human-derived NPPCs exhibited characteristics similar to BMSCs in their ability to form cell colonies, their rate of cell proliferation and stem cell-like gene expression, Liu et al. (2011) have found that human-derived CESCs could be induced into osteoblasts, adipocytes, and chondrocytes, and Feng et al. (2010) have shown that AFPC have the ability to differentiate into adipocytes, chondrocytes, osteoblasts, neurons and endothelial cells. Nevertheless, IVD progenitor cells also have different biological characteristics from other progenitor cells. In terms of differentiation ability, compared with MSCs, the NPPCs are better at chondrogenesis but worse at adipocyte differentiation (Blanco et al., 2010; Li X.C. et al., 2017), and the CESCs have more advantages in differentiation into osteogenesis and chondrogenesis (Liu et al., 2011). In terms of adapting to the microenvironment in IVD, IVD progenitor cells seem to have better performance. Tao et al. (2013) have found that NPPCs are well-able to resist environments with high permeability. Han et al. (2014) have revealed that NPPCs are more adaptable to acidic microenvironments than ADSCs. Moreover, Li et al. (2013) have indicated that NPPCs show greater levels of activity than ADSCs, undergoing more proliferation and exhibiting greater chondrogenic differentiation potential in hypoxic conditions.

In conclusion, the discovery of IVD progenitor cells not only confirms the hypotheses about the restoration of cell populations in organisms, but also provides potential methods for the treatment of IVD endogenous repair (Blanco et al., 2010; Becerra et al., 2011; Wang F. et al., 2015).

### Progenitor Cells for Homing

**FIGURE 2:**
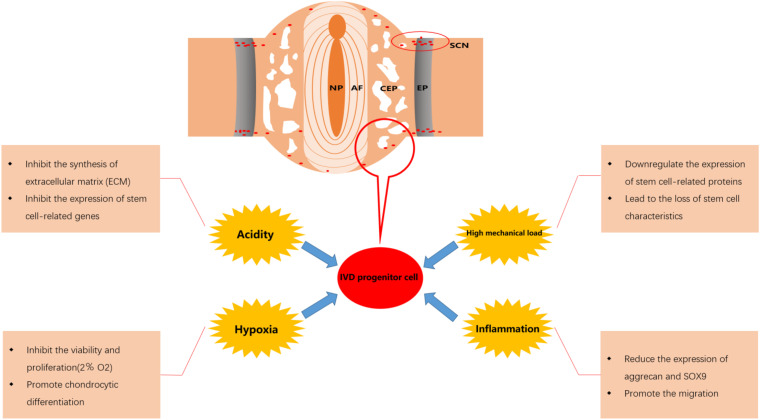
Effect of microenvironment on intervertebral disk (IVD) progenitor cells in degenerated IVD. NP, nucleus pulposus; AF, annulus fibrosus; CEP, cartilage endplate; EP, epiphyseal plate; SCN, stem cell niche.

It is clear that progenitor/stem cell-based therapeutics, whether in the form of exogenous or endogenous cells, require efficient induction of migration of sufficiently reparative cells to the target site (Laird et al., 2008). Progenitor cells homing is the process of the active recruitment of endogenous cells, including progenitor/stem cells, into a desired anatomic site for therapeutic applications (Chen et al., 2011). Interestingly, the stem cell niches of IVD are specialized anatomical regions surrounding the IVD (Figure 2), which play a role for sustaining/replenishing the progenitor cell pool and maintaining the IVD morphology and function (Henriksson et al., 2009). It has been reported that there are progenitor cells involved in the endogenous repair of IVD, including IVD progenitor cells, BMSC and the progenitor cells from stem cell niche (Li et al., 2015). In addition, we have previously reviewed the migration route of progenitor cell from stem cell niches into the disk (Ma et al., 2019). Compared with exogenous cell transplantation strategies, the endogenous repair pattern based on progenitor cell homing may have better biological safety and rationality, which are expected to overcome some of the limitations of exogenous cell transplantation and become an important supplement to IVD cell regeneration therapy.

## Endogenous Repair Strategies Based on IVD Progenitor Cells

When a tissue is damaged or degenerated, the normal pattern of endogenous repair is to recruit endogenous progenitor cells to the damaged or degenerated tissues through specific signals, where these recruited cells undergo proliferation and differentiation to accomplish self-repair and restoration of its structural organization (Forbes and Rosenthal, 2014). Xia et al. (2018) have reviewed the involvement of progenitor cells in endogenous repair, occurring principally via endocrine actions, proliferative differentiation and an immune mechanism. Unfortunately, although this endogenous repair mechanism is also present in the IVD, when the IVD is seriously injured, the repair effect of progenitor cells on IVD cannot meet the need for repair, which is the main reason leading to the failure of IVD endogenous repair (Ma et al., 2019). Actually, the activity and number of progenitor cells within IVD are affected by the adverse microenvironment in the damaged IVD. For instance, we have found that the number of endogenous progenitor cells migrated to IVD is affected by external pressure in the degenerative model of rabbit IVD under controlled axial pressure (Deng et al., 2013). Previous studies have also shown that as degeneration worsens, the number of progenitor cells within IVD decreases, combined with a decreased ability of endogenous progenitor cells to respond to signals of injury (Liang et al., 2018). In addition, we have demonstrated that a high mechanical load can cause apoptosis in NP cells via a caspase-dependent mitochondrial pathway (Ding et al., 2012) and advanced glycation end-products (AGEs) induce annulus fibrosus (AF) cell apoptosis also via the mitochondrial pathway (Hu et al., 2019). Accordingly, we speculate that the progenitor cells within IVD may also undergo a similar apoptosis process under AGES and high mechanical load.

In accordance with the studies on progenitor cells within IVD above, it can summarize as follows: first, the endogenous progenitor cells can be actively attracted to the sites where IVD is damaged or degenerative to participate in the repair. Secondly, there are mechanisms for regulating progenitor cells migration in IVD and these regulation mechanisms are also affected by microenvironment of IVD. Thirdly, the endogenous repair of IVD sometimes fails to effectively prevent the degeneration process of IVD as expected.

It is currently believed that the reason for the failure of IVD endogenous repair is mainly due to the insufficient number of endogenous progenitor cells recruited to the damaged site and the adverse microenvironment in degenerated IVD affecting the activity and viability of IVD progenitor cells (Ma et al., 2019). Therefore, promoting the migration of progenitor cells into the IVD, maintaining sufficient number of progenitor cells within the IVD and resisting the adverse effects of the microenvironment on progenitor may be the main strategies for the current research of endogenous repair based on progenitor cells.

### Promote the Migration of Progenitor Cells to IVD

The migration of endogenous progenitor cells to damaged tissues is a dynamic and complex regulatory process involving cytokines, intercellular interactions, intracellular signal transduction and interactions within intracellular structures (Xia et al., 2018). Previous studies of these process-related mechanisms have inspired us in enhancing the migration of IVD progenitor cells to IVD.

Using chemokines to recruit progenitor cells is a common method to promote IVD repair. A number of factors that induce chemotaxis have been identified, including interleukin-1β (IL-1β), tumor necrosis factor-α (TNF-α), monocyte chemoattractant protein-1 (MCP-1), stromal cell-derived factor-1 (SDF-1), insulin-like growth factor-1 (IGF-1), fibroblast growth factor-2, transforming growth factor-β (TGF-β), platelet-derived growth factor (PDGF), and macrophage-derived proteins, CCL5, *etc* (Grad et al., 2015; Nitzsche et al., 2017). These factors combine with corresponding receptors that result in the chemotactic migration of progenitor/stem cells. For instance, it has been reported that CCL5 is the main signal molecule involved in cell recruitment and its content increases in degenerative IVD. Interestingly, with the increase of CCL5 content, MSC significantly increase the expression of CCL5 receptors (CCR1 and CCR4) (Pattappa et al., 2014). It is worth mentioning that Frapin et al. (2020) recently successfully constructed a delivery system based on pullulan microbeads to sequentially release CCL5 and recruit IVD progenitor cells into NP tissue, followed by the release of the growth factors TGF-β1 and GDF-5, to complete the extracellular matrix remodeling.

As one of the key regulators of progenitor/stem cells, Small non-coding RNAs (miRNAs) are also involved in regulating the migration of a variety of cells (Gangaraju and Lin, 2009). It has been reported that a variety of miRNAs including miR-124 (Yue et al., 2016), miR-146a-5p (Hsieh et al., 2013), miR-26b, miR-221 (Zhu et al., 2016), miR-27b (Lü et al., 2012), miR-335 (Tomé et al., 2011), miR-375 (He et al., 2018) and miR-9-5p (Li X. et al., 2017) are involved in the migration of MSCs in different ways. It is a common way to improve the migration ability of cells by regulating the expression of miRNAs related to chemotactic function in cells. For instance, overexpression of miR-221 or miR-26b and inhibition of miR-124 in MSCs can enhance the migration ability of MSCs (Yue et al., 2016; Zhu et al., 2016). Therefore, promoting IVD progenitor cell migration through miRNAs with chemotactic function may also be a promising IVD endogenous repair strategy. In addition, miRNA combined with extracellular vesicles or biomaterials is also a popular choice. As a kind of extracellular vesicles actively secreted by cells, exosomes can carry active molecules such as cytokines, miRNA, and DNA into the recipient cells, thereby efficiently and targetedly regulating the biological behavior of the recipient cells (Simons and Raposo, 2009). For instance, it has been reported that MSCs deliver exogenous miR-21 via exosomes to inhibit NP cell apoptosis and reduce IVD degeneration (Cheng et al., 2018), and NP cells derived exosomes can promote the migration of MSC (Lan et al., 2019). Therefore, we speculate that the delivery of miRNAs related to migration to progenitor cells via exosomes to regulate the migration ability of progenitor cells may be a potentially promising method.

### Overcome the Adverse Effects of the Microenvironment on IVD Progenitor Cells

The effects of the fate of progenitor cells after migration on their repair function should also be taken into consideration. After the IVD progenitor cells migrate to the damaged IVD tissue, they will be affected by the adverse microenvironment in the IVD. Although IVD progenitor cells may show better adaptability to this adverse microenvironment and perform an important role in maintaining the balance of the IVD microenvironment, the activity and viability of IVD progenitor cells will still be significantly affected (Brisby et al., 2013; Han et al., 2014). Various factors such as hypoxia, low pH, high mechanical load and inflammation in degenerative IVD will result in reduced the migration of IVD progenitor cells and a decline in the number of IVD progenitor cells, which may be an important reason for the failure of endogenous repair (Tam et al., 2014; Zekri et al., 2015; Liu et al., 2017). Therefore, it is necessary to overcome the adverse effects of the microenvironment in degenerative IVD to maintain a sufficient number of IVD progenitor cells and enhance the efficacy of IVD progenitor cells in the treatment of degenerative IVD. In Figure 2, we displayed the effect of microenvironment on IVD progenitor cell in degenerated IVD.

#### Hypoxia

A number of studies have demonstrated that progenitor cells reside in hypoxic microenvironments, which can help progenitor cells maintain an undifferentiated phenotype (Muller et al., 2011; Stoyanov et al., 2011; Pei et al., 2012; Wang X.M. et al., 2015; Goncalves et al., 2017). For instance, hypoxia can effectively inhibit MSC senescence and maintains the characteristics of stem cells via down-regulation of E2A-p21 by HIF-TWIST (Tsai et al., 2011). However, it has been reported that severe hypoxia (oxygen tension <1%) will induce MSC apoptosis (Potier et al., 2007) and hypoxia (2% O_2_) significantly inhibits the viability and proliferation of NPPC, but promotes chondrocytic differentiation (Li et al., 2013). It can be inferred that different degrees of hypoxia have different effects on the biological activity and function of progenitor cells. Interestingly, it has been reported that the capability of MSCs to migrate *in vitro* decreases significantly in severe hypoxic microenvironments (1% O_2_) (Raheja et al., 2011), while their ability of proliferation and migration increases via transduction though the HIF-1a/FASN/mTORC1 pathway in slightly more oxygenated microenvironments (2.2% O_2_) (Lee H.J. et al., 2015). Accordingly, we speculate that different degrees of hypoxia may also affect the migration ability of IVD progenitor cells and there may be a range of oxygen concentration that is most suitable for mobilizing IVD progenitor cells to repair the IVD. Unfortunately, there are few reports on the regulatory mechanisms and protection strategies of IVD progenitor cells under hypoxia.

#### Acidity

The IVD is the largest avascular tissue in the human body, which only relies on CEP to exchange nutrients and metabolites. With the degeneration of IVD, the CEP is gradually calcified, which leads to the decrease of exchange and the accumulation of lactic acid in IVD cells (Agrawal et al., 2007; Loreto et al., 2011; Colombier et al., 2014). Normally, the mean pH within an IVD is 7.0–7.2, while the pH may decrease to as low as 6.5 in a severely degenerated IVD (Wuertz et al., 2009). It is worth noting that NPPC can resist the inhibitory effect of acidic microenvironment on cell activity and viability to some extent (Han et al., 2014). Nevertheless, NPPC will still be adversely affected by the acidic microenvironment of degenerative IVD. Studies have reported that the acidic microenvironment in degenerated IVD can reduce the expression of aggrecan, collagen II, metalloproteinase-3 (TIMP-3) and increase the expression of matrix metalloproteinase-2 (MMP-2), thrombospondin motifs-4 (ADAMTS-4) in NPPCs, which will cause the obstruction of ECM synthesis (Han et al., 2014; Liu et al., 2017). In addition, it has been reported that the acidic microenvironment in degenerative IVD can inhibit the expression of IVD stem cell-related genes, reduce the proliferation and increase the apoptosis of IVD progenitor cells (Liu et al., 2017).

In recent years, studies have reported that there is an extracellular receptor in IVD cells that responds to acidic pH: Acid-sensing ion channels (ASICs), which can be activated by acidosis, lactic acid or arachidonic acid (Xiong et al., 2004; Sun et al., 2013; Wang Y.C. et al., 2015). ASICs are categorized into six subtypes: ASIC1a, ASIC1b, ASIC2a, ASIC2b, ASIC3, and ASIC4. Compared with healthy IVD cells, the expression of ASICs is increased in degenerative IVD cells. Interestingly, the increased subtypes of ASICs in NP cells are ASIC1, ASIC2, and ASIC3, while those in AF cells are ASIC1 and ASIC4 (Yingjun and Xun, 2013; Cuesta et al., 2014). In addition, studies have demonstrated that ASIC3 can increase the tolerance of NP and AF cells to acidic microenvironment by increasing the expression of nerve growth factor (NGF), while ASIC1 is able to promote the apoptosis of CEP by mediating the influx of intracellular Ca^2+^ (Uchiyama et al., 2007; Li et al., 2014). Therefore, up-regulating the expression of ASIC3 or down-regulating the expression of ASIC1 in cells may be a potential way to protect IVD cells (Navone et al., 2012), and we speculate that this method can also protect IVD progenitor cells from the adverse effects of acidic microenvironment. Notably, it has been reported that amiloride can reduce the adverse effects of acid on NPPC by non-specifically inhibiting ASIC, which suggests that the acidic pH can regulate the biological activity of NPPCs via ASICs (Liu et al., 2017). Nevertheless, there are still few reports on the relationship between different IVD progenitor cells and ASIC subtypes and the mechanism by which ASIC regulates the metabolic activity of IVD progenitor cells in an acidic microenvironment, and further research is needed.

#### Mechanical Load

From a biomechanical point of view, all cells in IVD are exposed to mechanical load (Bowles and Setton, 2017). As degeneration occurs, the compressive force cannot be evenly distributed over the IVD, which can cause an increase in load and even acceleration of degeneration of the IVD (Smith et al., 2011; Hodges et al., 2015). We have demonstrated that microenvironments with continuous high mechanical loads in the IVD inhibit viability, migration, differentiation of NPPCs and lead to the loss of the characteristics of progenitor cells, which interferes with the endogenous repair of IVD (Liang et al., 2018). Due to the variability of mechanical load, it becomes relatively difficult to protect IVD cells from damage caused by mechanical load. Yuan et al. (2018) have reported that BNIP3 (adenovirus E1B 19 kDa interacting protein 3) contributed to the regulation of cyclic stretch-induced apoptosis of CEPC in an *in vitro* model. We have also explored some methods to overcome mechanical load. For instance, we have found that the edaravone can ameliorate compression-induced apoptosis in NP cells by inhibition of ROS production, blocking the collapse of anti-mitochondrial membrane potential and inhibition of increased intracellular Ca^2+^ (Lin et al., 2017), and the cyclosporine A (CsA) can effectively inhibit compression-induced apoptosis of NPPCs by mitigating mitochondrial dysfunction and oxidative stress (Li et al., 2018). Interestingly, we have also found that moderate compression is able to increase cell viability (Liang et al., 2018). Besides, it has been reported that cyclic mechanical load is beneficial to the differentiation of NPPCs into mature NPCs (Wang F. et al., 2015). We speculate that mechanical load may be used as a starting factor to initiate endogenous repair at an early stage, but after a period of time, mechanical load may be one of the reasons for the failure of endogenous repair. If we can find the time node at which the endogenous repair failure starts and take timely treatment of the injured IVD before that, it may be able to reverse the failure of endogenous repair.

#### Inflammation

Previous studies have shown that the expression levels of inflammatory factors interleukin (IL)-1α/β, IL-6, IL-17, and TNF-α in degenerated IVD will be significantly increased. These factors not only cause the apoptosis of IVD cells, but also degrade the extracellular matrix through upregulation of catabolic factors [such as matrix metalloproteinases (MMP) and a disintegrin—like and metalloproteinase with thrombospondin motifs (ADAMTS)], which will eventually destroy the balance between repair and damage of IVD (Millward-Sadler et al., 2009; Risbud and Shapiro, 2014). It is worth noting that many Inflammatory factors can also increase the expression level of chemokines in IVD (Risbud and Shapiro, 2014). For instance, as mentioned in the 4.1, CCL5, which is considered to be a key chemokine produced by degenerative IVD, has been proven to promote progenitor cell migration (Pattappa et al., 2014). It has been reported that IL-1β and TNF-α can significantly up-regulate the expression level of CCL5 in IVD (Kepler et al., 2013). This suggests that inflammatory factors, like the mechanical load mentioned above, may also be involved in maintaining the balance between IVD repair and injury in the early stage of IVD degeneration. However, less is known about the impact of IVD inflammatory environment on IVD progenitor cells. It has been demonstrated that IL-1β, as an inflammatory factors positively correlated with the severity of IVD degeneration, can not only inhibit the proliferation of NPPCs, but also reduce the expression of aggrecan and SOX9 by NPPCs (Ning et al., 2014; Lyu et al., 2019). There have been many reports on the strategy of inhibiting the release of inflammatory factors to alleviate IVD degeneration, such as melatonin (Zhang et al., 2019), MSC-exosomes (Xia et al., 2019), glycyrrhizin (Liu X. et al., 2019), aspirin (Liu Y. et al., 2019), etc. Therefore, reducing the production of inflammatory factors in IVD may be used as an effective strategy to protect IVD progenitor cells.

#### Effective Biological Factors Delivery Strategies

Recruiting progenitor cells by injecting chemokines into IVD to drive endogenous repair is a promising strategy. Unfortunately, these biological factors usually have a short half-life and require repeated injections to achieve the desired therapeutic effect, which may increase the risk of IVD degeneration (Masuda, 2008). In addition, the activity of progenitor cells that migrate to the damaged tissue will also be affected by the local microenvironment (Han et al., 2014). In this context, the development of an effective drug delivery platform is of great significance for the treatment of IVD.

Formulation of tissue engineering scaffold materials that are biocompatible and possess suitable biological activity provides an excellent strategy for protecting the IVD progenitor cells, which is an important factor in the effective treatment of IVD degeneration (Thorpe et al., 2017). Due to its biocompatibility, injectability, sustained-release function and similar components to ECM, bio-hydrogels are of great significance for IVD application. Moreover, bio-hydrogels can provide mechanical support and 3D microenvironment suitable for the survival, proliferation and differentiation of reparative cells (Flégeau et al., 2017). For instance, comparing with using SDF-1 alone, topical use of HAP [a thermoreversible hyaluronan-poly(N-isopropylacrylamide)] hydrogels containing SDF-1 can promote the migration of MSCs into degenerated IVD and improving their therapeutic efficiency better (Pereira et al., 2014).

Based on the above related studies on IVD progenitor cell repair, we can infer that ideal materials may generally require the following: (1) Provision of a progenitor cell migration channel scaffold; (2) Excellent sustained release; (3) Maintaining sufficient number of progenitor cells within the IVD; and (4) Assistance for IVD progenitor cells to resist the poor microenvironment of IVDs. Therefore, biomaterials are also a field worthy of our attention in terms of regenerative therapies based on endogenous cell migration.

## Explore Potential IVD Repair Strategies From the Perspective of Intercellular Communication

Intercellular communication is an important way of intercellular interaction. Common mechanisms of intercellular communication include soluble factors (such as growth factors, neurotransmitters, and cytokines/chemokines), gap junctions, exosomes and tunnel nanotubes (TNTS), etc (Murray and Krasnodembskaya, 2019). It has been early reported that notochordal cells participates in IVD repair through paracrine action (Erwin, 2008) and the inflammatory factors can lead to the damage of cells in IVD (Risbud and Shapiro, 2014). With the development of regenerative medicine, the exploration of cell regeneration therapy has extended to the level of organelles and even small molecules. In recent years, intercellular communication mediated by the transfer of cytoplasmic substances and organelles has aroused more and more attention (Murray and Krasnodembskaya, 2019). For instance, it has been shown that mitochondria can transport from MSC to ocular cells through TNTs (an open channels between cells) to achieve the purpose of treatment (Jiang et al., 2020). It can be speculated that similar repair phenomena may also exist in IVD, which will be a promising research direction.

As the main subtype of extracellular vesicles, exosomes play an important role in intercellular communication. Exosome derived from different tissues not only has its specific protein molecules, such as surface markers, but also contains its functional bioactive molecules, including cytokines, growth factor receptors, liposomes, RNA and so on (Zhang et al., 2016). It has been reported that progenitor cell-derived exosomes have the repair properties of progenitor cells, which can realize cell-free therapy and avoid some ethical problems that may arise from cell therapy (Phinney and Pittenger, 2017). As a research hotspot, exosomes have also been reported in the field of IVD degeneration research in recent years.

Notably, exosomes can be used as a “substitute” for progenitor cells to promote tissue repair by regulating mature cells. For instance, it has been reported that exosomes derived from MSC (MSC-exos) can stop the progression of degeneration of IVD *in vivo* by modulating endoplasmic reticulum stress (Liao et al., 2019). In addition, it has been shown that MSC-exos could prevent the progression of degenerative changes of IVD through suppressing NLRP3 inflammasome activation and inflammatory mediators (Xia et al., 2019).

Exosomes can also be used as a “messenger” for mature cells to participate in the regulation of progenitor cell functions. For example, the NP-derived exosomes (NP-exos) can promote MSC migration and differentiation into NP-like cells *in vivo* (Lu et al., 2017; Lan et al., 2019). Whether NP-exos act on endogenous progenitor cells have the same phenomenon as on MSCs remains to be further studied.

In summary, there are two important issues that need to be elucidated in IVD degeneration research. The first is how to activate IVD progenitor cells within IVD and recruit endogenous progenitor cells from the IVD niches and vertebrae bone marrow through intercellular communication. The second is why this endogenous repair fails. If the endogenous repair of IVD can be promoted by regulating the communication mechanism between cells, it will help to achieve *in situ* regeneration of IVD tissue and bring new strategies to the research of IVD degeneration biological treatment.

## Conclusion and Prospects

The discovery of IVD endogenous progenitor cells brings new solutions for the biological cell therapy of IVD degeneration. Inducing IVD progenitor cells to migrate to the damaged IVD tissues and maintaining the activity and viability of IVD progenitor cells are the main directions of current IVD endogenous repair research. But so far, there are still few studies on the mechanism of IVD endogenous repair. The relationship between IVD progenitor cells and damaged IVD tissues/cells and between IVD progenitor cells and IVD microenvironment needs further clarification. In addition, Intercellular interaction is a research area worthy of attention. The research on the biological information exchange between IVD progenitor cells and the damaged tissues/cells may further deepen our understanding of the IVD endogenous repair.

## Author Contributions

YZ wrote the article and reviewed and/or edited the manuscript. LX and XC designed the study. YH, WW, ZG, and FY researched data and discussed the results on the manuscript. All authors contributed to the article and approved the submitted version.

## Conflict of Interest

The authors declare that the research was conducted in the absence of any commercial or financial relationships that could be construed as a potential conflict of interest.
